# Mitochondrial‐targeted catalase is good for the old mouse proteome, but not for the young: ‘reverse’ antagonistic pleiotropy?

**DOI:** 10.1111/acel.12472

**Published:** 2016-04-08

**Authors:** Nathan Basisty, Dao‐Fu Dai, Arni Gagnidze, Lemuel Gitari, Jeanne Fredrickson, Yvonne Maina, Richard P. Beyer, Mary J. Emond, Edward J. Hsieh, Michael J. MacCoss, George M. Martin, Peter S. Rabinovitch

**Affiliations:** ^1^ Department of PathologyUniversity of Washington1959 NE Pacific AveSeattleWA98195USA; ^2^ Department of Environmental HealthUniversity of Washington1959 NE Pacific AveSeattleWA98195USA; ^3^ Department of BiostatisticsUniversity of Washington1959 NE Pacific AveSeattleWA98195USA; ^4^ Department of Genome SciencesUniversity of Washington1959 NE Pacific AveSeattleWA98195USA; ^5^ Present address: Merck901 California AvePalo AltoCA94304USA; ^6^ Present address: The Buck Institute for Research on Aging8001 Redwood BoulevardNovatoCA94945USA

**Keywords:** aging, mitochondria, catalase, antagonistic pleiotropy, protein turnover, reactive oxygen species

## Abstract

Reactive oxygen species (ROS) are highly reactive oxygen‐containing molecules associated with aging and a broad spectrum of pathologies. We have previously shown that transgenic expression of the antioxidant enzyme catalase targeted to the mitochondria (mCAT) in mice reduces ROS, attenuates age‐related disease, and increases lifespan. However, it has been increasingly recognized that ROS also has beneficial roles in signaling, hormesis, stress response, and immunity. We therefore hypothesized that mCAT might be beneficial only when ROS approaches pathological levels in older age and might not be advantageous at a younger age when basal ROS is low. We analyzed abundance and turnover of the global proteome in hearts and livers of young (4 month) and old (20 month) mCAT and wild‐type (WT) mice. In old hearts and livers of WT mice, protein half‐lives were reduced compared to young, while in mCAT mice the reverse was observed; the longest half‐lives were seen in old mCAT mice and the shortest in young mCAT. Protein abundance of old mCAT hearts recapitulated a more youthful proteomic expression profile (*P*‐value < 0.01). However, young mCAT mice partially phenocopied the older wild‐type proteome (*P*‐value < 0.01). Age strongly interacts with mCAT, consistent with antagonistic pleiotropy in the reverse of the typical direction. These findings underscore the contrasting roles of ROS in young vs. old mice and indicate the need for better understanding of the interaction between dose and age in assessing the efficacy of therapeutic interventions in aging, including mitochondrial antioxidants.

## Introduction

Reactive oxygen species (ROS) are associated with the progression of a broad spectrum of pathologies including aging (Harman, 1956), neurodegeneration, metabolic syndrome (Fisher‐Wellman & Neufer, 2012; Dodson *et al*., 2013), heart disease (Dai *et al*., 2013; Wohlgemuth *et al*., 2014), cancer, and others (Brieger *et al*., 2012). Mechanistically, this has largely been attributed to oxidative modification of cellular macromolecules, including lipids (Arai, 2014), DNA (Lee *et al*., 2010a), and proteins (Forman *et al*., 2014). While ROS have been widely regarded as a major component of aging since the ‘free radical theory of aging’ was proposed in the 1950s (Harman, 1956), there is an increasing appreciation that ROS also serve important physiological signaling roles. It is therefore important to closely examine both negative and positive consequences of therapeutic interventions that target ROS.

Given that oxidative modifications can impair the activity of macromolecules, and the well‐documented correlation between oxidative damage and aging reported in almost all models studied, it has been tempting to conclude that this is a likely mechanism for aging (Bokov *et al*., 2004). However, there are many observations at odds with this theory of aging. Clinical trials of dietary antioxidants to reduce cancer (Goodman *et al*., 2011), gastrointestinal (Bhardwaj, 2008), neurological (Mecocci & Polidori, 2012), rheumatoid (Jalili *et al*., 2014), endocrine (Golbidi & Laher, 2010), and cardiovascular diseases (Kris‐Etherton *et al*., 2004) have thus far shown little to no efficacy. Some have shown adverse outcomes (Bjelakovic *et al*., 2012). In mice, deletion of many antioxidant enzymes has little effect on lifespan and, importantly, overexpression of several antioxidants including superoxide dismutase and peroxisomal catalase has failed to extend lifespan (Pérez *et al*., 2009). In fact, mice lacking the cellular antioxidant glutathione peroxidase 1 are protected from high‐fat‐induced insulin resistance, and this benefit is lost upon the administration of an antioxidant (Loh *et al*., 2009). Compared to mice with a median lifespan just over 2 years, the naked mole rat shows remarkable longevity, living 10–30 years, in spite of similar rates of ROS production and more extensive oxidative damage to its tissues over its lifetime (Lewis *et al*., 2013), suggesting that ROS may not necessarily play a causative role in aging. Additionally, while mtDNA mutations increase with age, the characteristic mutations created by ROS are not among those most seen by the new method of duplex sequencing, suggesting that ROS may not be a driver of somatic mutations in aging (Itsara *et al*., 2014). In C elegans, an invertebrate model of aging, several strains deficient in respiratory proteins that produce excess reactive oxygen species are in fact longer lived than WT controls (Lee *et al*., 2010b), and ROS has been shown to be indispensable for the increased lifespan of glycolysis‐deficient worms or worms on glucose restriction (Schulz *et al*., 2007).

Our group has previously shown that mice overexpressing mitochondrial‐targeted catalase (mCAT), but not nuclear or peroxisomal catalase, have an approximately 20% increased median and maximal lifespan (Schriner *et al*., 2005), suggesting that reducing ROS specifically in the mitochondria is key to achieving a beneficial effect on aging. mCAT has been shown to reduce oxidative modification of DNA and proteins and delays the progression of multiple pathologies (Treuting *et al*., 2008). We have also demonstrated that mCAT is protective against cardiac aging (Dai *et al*., 2009), as well as models of cardiac hypertrophy and failure in young mice (Dai *et al*., 2012), (Dai *et al*., 2011). Other laboratories have demonstrated that mCAT mice have additional benefits, such as improved amyloid precursor protein processing (Mao *et al*., 2012), reduced loss of bone after estrogen withdrawal (Bartell *et al*., 2014), reduced insulin resistance (Lee *et al*., 2010a), and protection against radiation exposure (Parihar *et al*., 2015). Similar benefits have been seen in mice treated pharmacologically with the mitochondrial‐targeted tetrapeptide SS‐31 (Dai *et al*., 2013) (d‐Arg‐2′, 6′‐dimethyltyrosine‐Lys‐Phe‐NH_2_), also known as Bendavia, which targets mitochondrial inner membrane cardiolipin, enhances electron‐transfer efficiency, and thereby reduces ROS production (Szeto, 2014). This report describes an analysis of global proteomic changes resulting from mCAT overexpression in the context of aging, measuring both protein abundance and turnover rates. We report changes in the hepatic and cardiac proteomes with age, comparing wild‐type and mCAT animals.

## Results

### mCAT has opposite effects on hepatic and cardiac protein turnover in young vs. old mice

To comprehensively quantify *in vivo* changes in global proteome half‐lives (HLs), we performed stable isotope metabolic labeling of mice by administering a synthetic diet containing ^2^H_3_‐leucine over a period of 17 days, as previously described (Karunadharma *et al*., 2015) (Fig. S1). Hearts and livers were collected at four subsequent time points from each of four experimental groups: young WT (YWT) and young mCAT (YmCAT) at 3–4 months of age, old WT (OWT) and old mCAT (OmCAT) at 18–21 months of age. All treatment groups contained a total of *n* = 11–12 biological replicates, with a total of *n* = 44–48 per tissue, which were analyzed by nLC‐MS/MS. Topograph software was used to estimate the proportion of newly synthesized protein at each time point, corrected for the enrichment of heavy leucine in the amino acid precursor pool (Hsieh *et al*., 2012), and HLs were calculated based on first‐order regressions of percentage of newly synthesized protein over time. We identified 11, 240 unique peptides mapping to 2,570 proteins in heart and 11, 461 unique peptides mapping to 3,145 proteins in liver. Raw data can be accessed in the public Chorus repository (see Materials and methods).

There were no significant protein abundance changes over the 17‐day labeling period in either the hearts or livers of mCAT and WT mice at either age (Fig. S2). This indicates that (i) the presence of multiple peptide isotopic peaks, which arise from the combination of heavy and light leucine in the diet, does not seriously alter the integration of peak area by Topograph to quantitate protein abundance; (ii) unchanging protein abundance suggests that the rates of protein synthesis and degradation are at steady states, as expected in adult animals.

**Figure 1:**
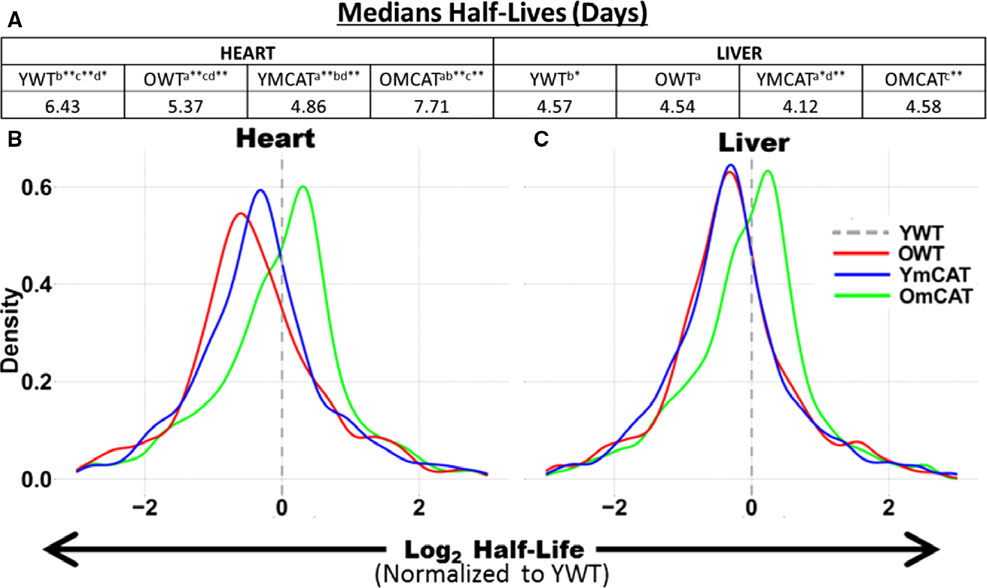
Distributions of the changes in proteome half‐life in (A) heart and (B) liver tissues by treatment—OWT, YmCAT, and OmCAT—each relative to young WT controls. Dotted lines mark the location of a ratio equal to one (YWT).

Median protein half‐lives are longer in heart than in liver, even among proteins common to both. The protein HLs in YWT hearts were 1.4 times longer (6.4 days) than in the liver (4.57 days) (Fig. 1A). In the heart, there was a significant difference in median half‐life between any two age/treatment groups; the longest half‐life was seen in OmCAT (7.71 days) followed by YWT (6.43 days), OWT (5.37 days), and YmCAT (4.86 days). The liver followed this same order over a smaller range, with half‐lives of 4.58, 4.57, 4.54, and 4.12 days in OmCAT, YWT, OWT, and YmCAT respectively. Histograms of the distribution of half‐lives in each experimental group in heart and liver are shown in Fig. S3. A more sensitive analysis was performed by normalizing HLs of each protein in each treatment group to the corresponding protein's YWT HL. This paired analysis of the protein‐by‐protein fold change in HL in each group vs. YWT is visualized in histograms for heart (Fig. 1B) and liver (Fig. 1C). A positive number indicates an increase in half‐life vs. YWT (in units of log_2_ fold change), while a negative number indicates a reduced HL relative to YWT. As previously reported (Dai *et al*., 2014; Karunadharma *et al*., 2015), old WT mice had significantly decreased HLs compared to young WT mice; however, old mCAT HLs were longer than old WT and thus more similar to young WT, consistent with restoring a more youthful proteome. Interestingly, the young mCAT had *shorter* HLs than YWT, and the effect of aging on mCAT protein half‐life *is thus a complete reversal of the change seen in WT mice*.

### Analysis of cardiac proteome changes in half‐life

**Figure 2:**
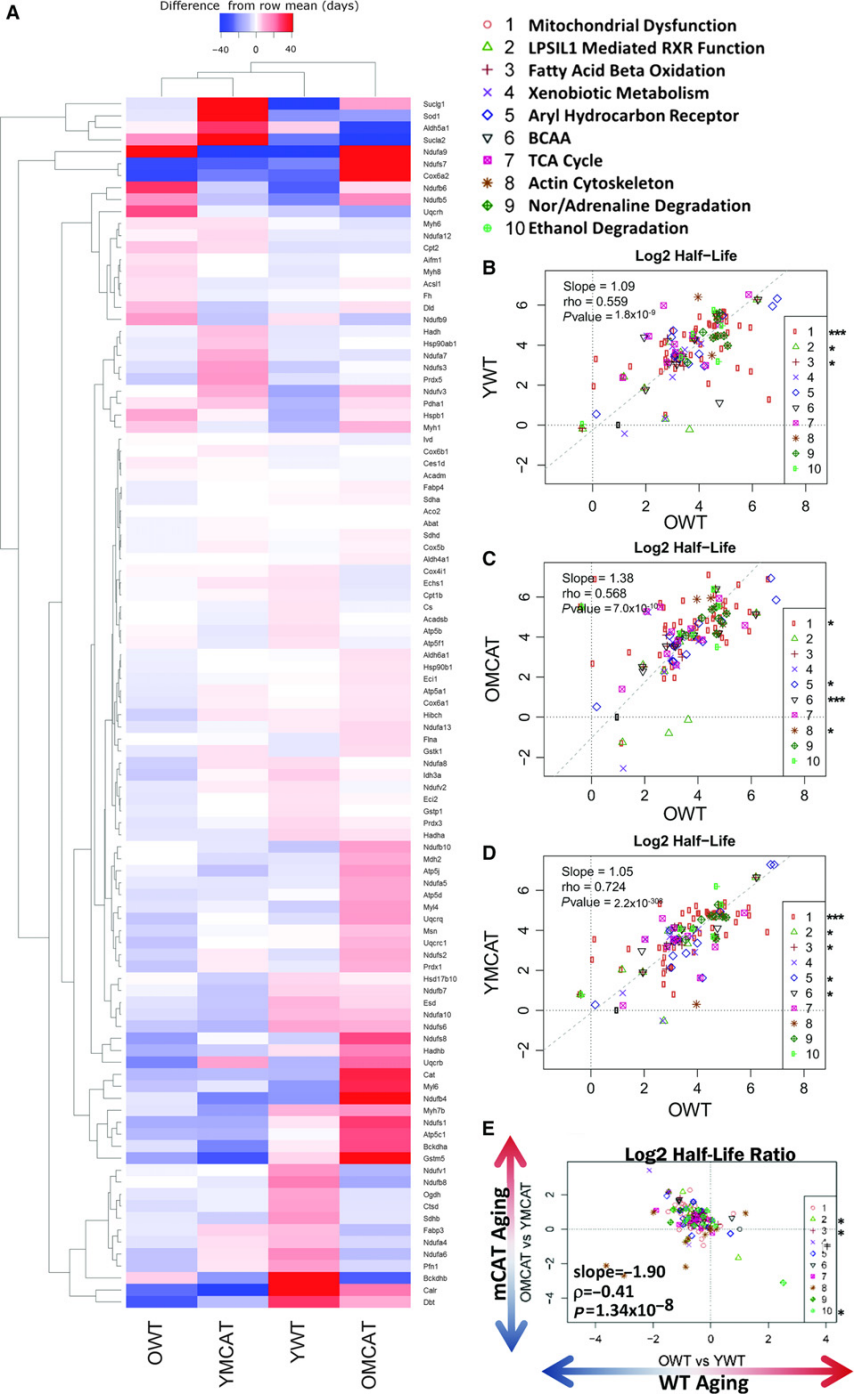
Hierarchical heatmap of all cardiac proteins significantly changed in half‐life during aging and correlation plots of cardiac proteins that significantly changed with age. (A) Heatmap depicting all proteins with significantly altered (*P*‐value < 0.05) in the statistical comparison of YWT and OWT proteome half‐lives. The four columns correspond to the treatment groups used in this study: YWT, OWT, YmCAT, and OmCAT. Colors in the heatmap represent the half‐life of a protein relative to its mean half‐life (in days) across all treatment groups (difference from row mean). Correlations were made between OWT half‐lives vs. each treatment group—(B) YWT, (C) OmCAT, and (D) YmCAT. (E) A correlation of WT aging (OWT/YWT) vs. mCAT aging (OmCAT/YmCAT). * *P*‐value < 0.05, **P‐value < 0.001 for Spearman's correlation of the individual pathways in the regressions.

Proteins that showed a significant age or mCAT‐dependent change in HL (*P*‐value < 0.05) were plotted on a heatmap with hierarchical clustering in both rows and columns (Fig. 2A). The first two columns, showing mostly decreases (blue) in half‐life of OWT and YmCAT compared to the row means, illustrate the visual similarity between aged WT and young mCAT mice. This is in contrast to the increasing half‐lives (red) seen in a higher proportion of proteins in the third and fourth columns, which include old mCAT and young WT mice.

The effects of mCAT overexpression on protein HLs were correlated with WT aging changes by bivariate plots for proteins in the top ten Ingenuity Pathway Analysis (IPA) pathways, ranked by significance of half‐life changes in WT aging (OWT vs. YWT) (Fig. 2B–D). The majority of the ‘top’ pathways were metabolic pathways (including mitochondrial dysfunction, xenobiotic metabolism, fatty acid oxidation, and TCA cycle) or signaling pathways (aryl hydrocarbon receptor signaling) and a few other notable pathways, such as actin cytoskeleton, which are closely tied to heart function. The regression in all top pathways combined confirms that the half‐lives of these top pathway proteins in young mCAT mice are more similar to those of old WT mice (slope = 1.05, *r* = 0.724, *P*‐value < 2.2 × 10^−308^, Fig. 2D) and lower than those of young WT (slope = 1.09, *r* = 0.559, *P*‐value = 1.8 × 10^−9^, Fig. 2B) or old mCAT (slope = 1.38, *r* = 0.568, *P*‐value = 7.0 × 10^−10^, Fig. 2C). A direct comparison of WT aging (OWT vs. YWT) to mCAT aging (OmCAT vs. YmCAT) also showed a strong negative correlation (Fig. 2D), highlighting the opposing effects of mCAT in young vs. old mice. All cardiac proteins and corresponding turnover values can be found in Data S1.

### Analysis of hepatic proteome changes in half‐life

**Figure 3:**
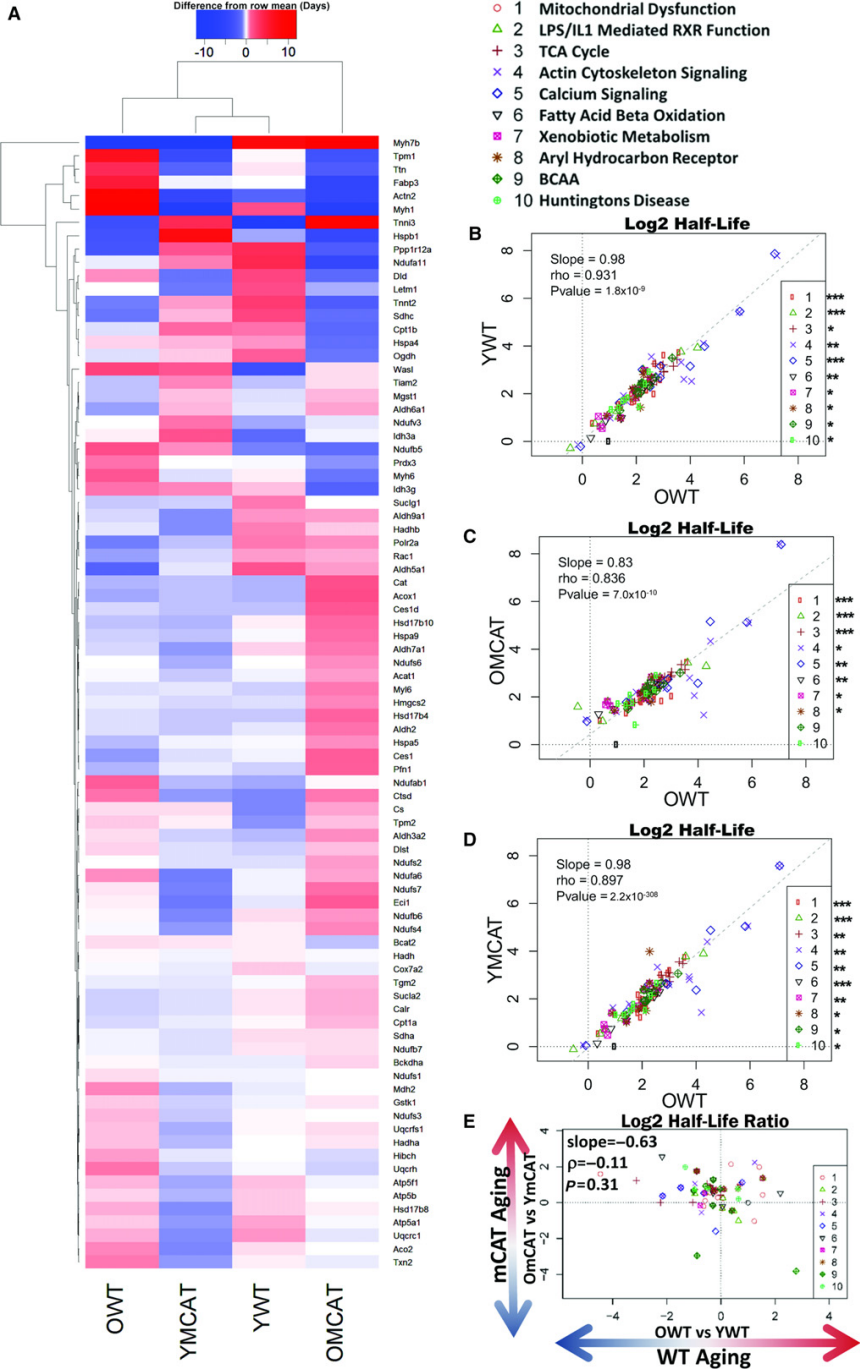
Heatmap and correlation plots of hepatic protein half‐lives that significantly changed with age. (A) Heatmap depicting all significantly changed (*P*‐value < 0.05) protein HLs during aging (OWT vs. YWT). The heatmap colors represent the half‐life of a protein relative to its mean half‐life (in days) across all treatment groups (difference from row mean). (B–D) Correlations of OWT half‐lives vs. (B) YWT, (C) OmCAT, and (D) YmCAT half‐lives. (E) A correlation of WT aging (OWT/YWT) vs. mCAT aging (OmCAT/YmCAT). ǂ*P*‐value < 0.10, * *P*‐value < 0.05, **P‐value < 0.001 for Spearman's correlation of the individual pathways in the regressions.

Hepatic proteome turnover partially recapitulated the effects seen in the heart (Fig. 3A), and the list of top affected pathways was highly overlapping in the two tissues (Fig. 3B–D). All treatment groups showed a highly significant degree of correlation with OWT in all ten top pathways combined, and this correlation remained significant for every individual pathway in YWT and YmCAT (Fig. 3B,D) as well as eight of ten of the pathways in OmCAT (Fig. 3C). This high degree of similarity between treatment groups in liver suggests that both aging and mCAT changes in half‐life are modest. Even so, the apparent visual differences between groups in the heatmap are consistent with the differences in the strength of these correlations. For example, OmCAT is the most divergent group in both cases (Fig. 3A,C). Additionally, mCAT aging did not show a significant correlation with WT aging (Fig. 3D). All hepatic proteins and corresponding turnover data can be found in Data S2.

### Protein abundance changes in the cardiac and hepatic proteomes

**Figure 4:**
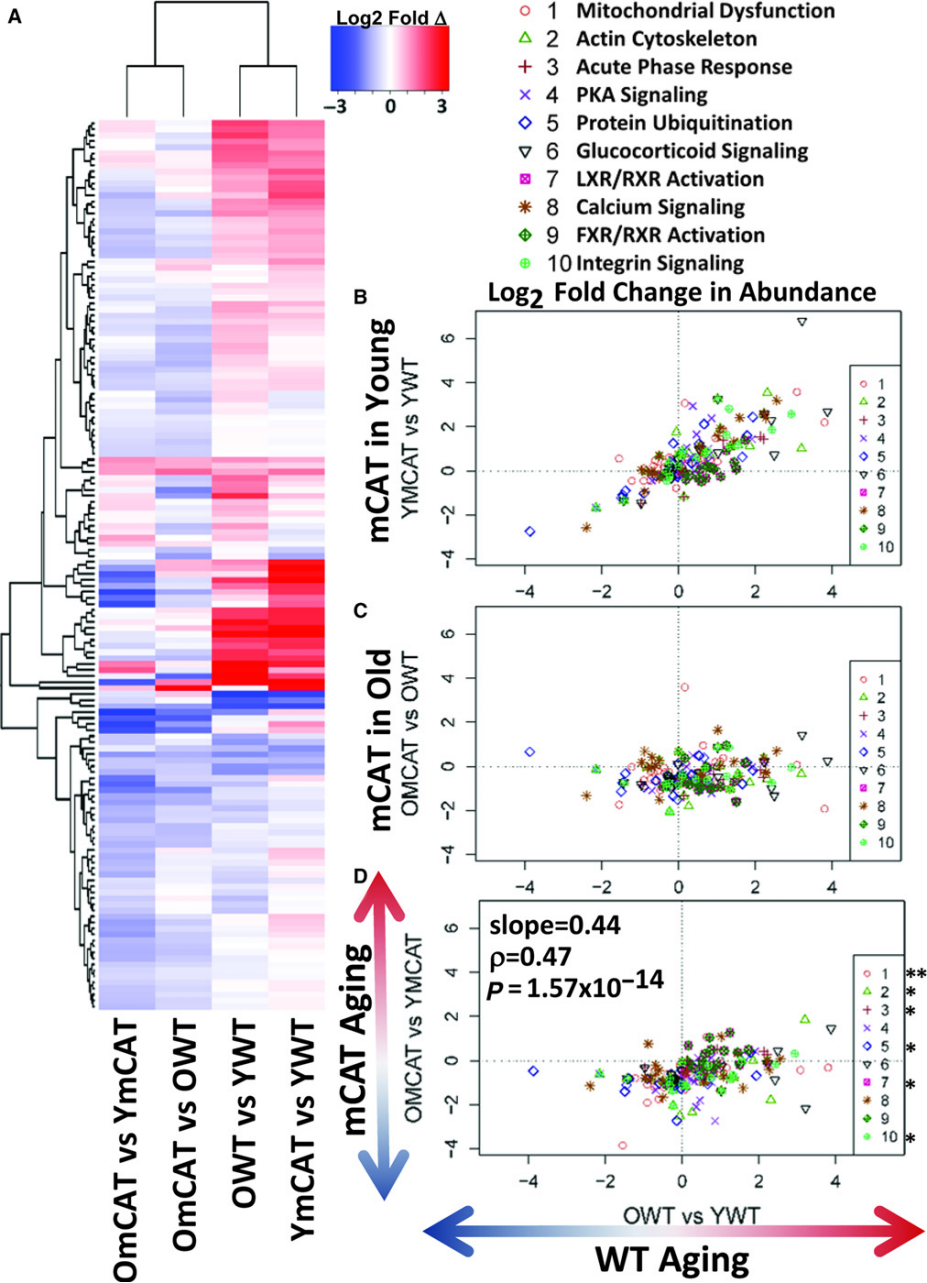
Heatmap of all significant cardiac protein abundance changes with age and correlations of WT aging to mCAT aging. (A) The heatmap shows the direction and magnitude of abundance changes between the groups noted in the column labels. (B–D) Correlation plots of changes in HL of proteins in the top ten IPA pathways, (B) The effect of mCAT in young mice (YmCAT vs. YWT) positively correlates with WT aging (OWT vs. YWT,* P*‐value < 0.05). (C) The effect of mCAT in old mice (OmCAT vs. OWT) had little to no correlation with WT aging. (D) The effect of aging in mCAT (OmCAT vs. YmCAT) vs. WT aging (OWT vs. YWT) shows a significant negative correlation, with a correlation coefficient of −0.5. * *P*‐value < 0.05, **P‐value < 0.001 for Spearman's correlation of the individual pathway between the x‐ and *y*‐axis groups.

Relative abundance data and statistics for the heart and liver proteomes can be found in Data S1 and Data S2, respectively. MS1 peak areas were calculated in Topograph and used to perform relative quantification of protein abundance (see Materials and methods). An inherent limitation to MS quantitation is that the intensity of peak areas is highly dependent on peptide ionization efficiency, which is very variable across peptides, and therefore provides no information about the absolute quantity of a peptide or protein. It is common to instead measure the relative change in any given peptide across samples. To compare the effects of mCAT and aging on protein abundance, we therefore examined the relative differences in peak area by examining the ratios of treatment groups: OWT/YWT (WT aging), OmCAT/YmCAT (mCAT aging), YmCAT/YWT, and OmCAT/OWT (the effects of mCAT expression in young and old mice). Our results show that, similar to the trend seen in half‐lives, the effects of mCAT in young mice (YmCAT vs. YWT, column 1) are clustered with WT aging effects (OWT vs. YWT, column 2), while mCAT aging changes (OmCAT vs. YmCAT, column 3) and mCAT effects in old mice (OmCAT vs. OWT, column 4) cluster with each other and often show an opposite pattern of expression to the first two columns (Fig. 4A). Because comparisons between sets of ratios with common terms (i.e., OWT/YWT vs YmCAT/YWT and OWT/YWT vs OmCAT/OWT) can introduce ‘spurious correlation’ (Pearson, 1987), we performed statistical analysis using partial correlations controlling for the common terms (Michael Lynn, 1992). Notably, mCAT expression levels in young mice tend to be in the same direction as OWT, as can be seen from the positive correlation in Fig. 4B (*P*‐value < 0.05). In contrast, mCAT effects in old mice (OmCAT vs OWT) were not correlated with WT aging changes, while mCAT aging (OmCAT vs YmCAT) was correlated with WT aging changes (Fig. 4D, *P*‐value = 1.57 × 10^−14^). Of the top 20 pathways most significantly changed in the heart abundance proteome (Fig. 4B–D), eight were in common with pathways most changed in the turnover dataset. Three of these top 20 pathways are protein turnover mechanisms: protein ubiquitination, p70s6K, and clathrin‐mediated endocytosis.

The effects of mCAT and aging on liver abundance appear to share many trends with heart changes, but to a much lesser extent (Fig. S4A–D). Again, aging effects (OWT vs. YWT) and the effects of mCAT in young mice (YmCAT vs. YWT) clustered as a pair in the heatmap, while mCAT aging (OmCAT vs YmCAT) and the effects of mCAT in old mice (OmCAT vs. OWT) were paired in another cluster (Fig. S4A). Young mCAT effects were significantly correlated with aging effects, albeit much more weakly than in heart (*P*‐value < 0.05). Of the top ten IPA pathways, the three that showed individually significant correlations were protein turnover pathways: EIF2 signaling, p70S6K signaling, and eiF4/p70S6K signaling. The effects of mCAT expression in old livers did not have a significant negative correlation with aging changes (Fig. S4C). However, the comparison of WT aging and mCAT aging effects (Fig. S4D) did show a small negative correlation (*P*‐value < 0.01).

**Figure 5:**
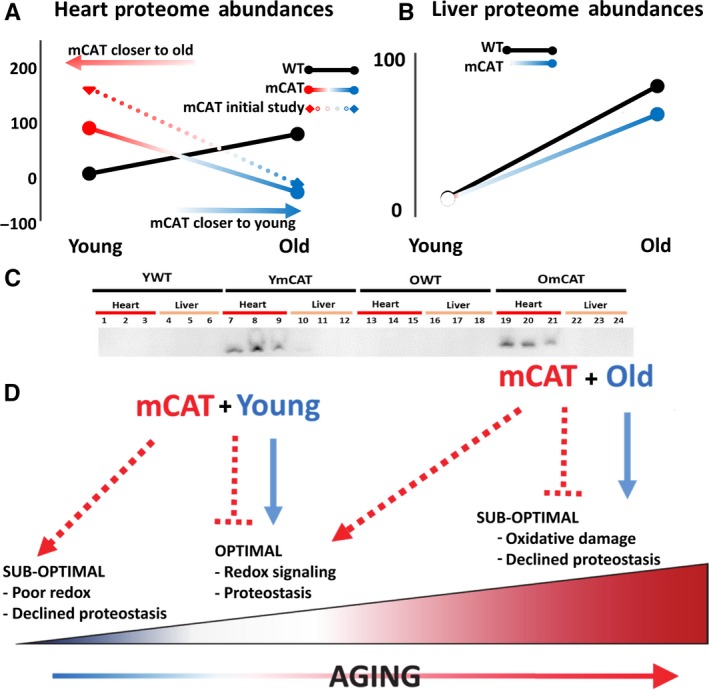
Reverse antagonistic pleiotropy in the proteome as a result of mCAT. We condensed proteomic changes into simple line plots depicting the trajectory of WT aging (black line) and mCAT aging (colored line). (See the Materials and methods for more detail.) (A) The heart proteome demonstrates reverse antagonistic pleiotropy: The YmCAT proteome more closely resembles old mice, while the OmCAT mouse is shifted toward a younger proteome compared to their OWT counterparts. (B) This effect was not seen in liver tissue, which (C) did not express mCAT protein, as measured by Western blotting and confirmed by qPCR (Fig S5). (D) Illustration depicting a model in which mCAT may be good in old mice, but not in young mice.

The significantly weaker mCAT effects in liver observed for both half‐lives and abundance are likely due to the much lower levels of liver mCAT expression from the chick beta actin‐promoted transgene. In contrast to highly expressed mCAT in heart, skeletal muscle, and brain, we have previously reported no difference from littermate controls in mCAT liver expression (Schriner *et al*., 2005). To verify these previous observations, we measured protein levels of mCAT by Western blot (Fig. 5C, S5) and mRNA expression by qPCR (Fig. S5); the results confirmed that mCAT protein and mRNA expressions are essentially undetectable in liver. This suggests either that undetectably small levels of mCAT expression in livers are sufficient to produce modest and partial effects in proteome abundance and turnover or that that the changes in liver are a cell non‐autonomous effect of mCAT expression in other tissues.

### mCAT aging exhibits reverse antagonistic pleiotropy

To compare the trajectories of wild‐type aging and mCAT aging, we condensed the proteomic changes into an index of aging change by taking the average absolute magnitude of the fold changes in protein abundance from YWT to OWT over all significantly altered (*q*‐value < 0.05) proteins in WT aging. By this metric, WT aging is an average 5.66 fold change in heart proteome abundance. For each of the same proteins, the YWT vs. YmCAT or YWT vs. OmCAT was similarly calculated and the average absolute change expressed as a percentage of the WT aging effect (Fig. 5). Consistent with the abundance correlations noted above, mCAT mice have a striking reversal of the WT aging trajectory; the YmCAT proteome index for these same proteins is nearly 100% of OWT in magnitude, but declines to YWT levels in the old mCAT proteome. This surprising result was initially seen in a separate ‘pilot’ experiment without heavy leucine labeling (Fig. 5B, dotted line, heatmap summary in Fig. S6). Some of these trends in significantly changed proteins in the aging cardiac proteome were further validated by Western blot (Fig. S7A,B). A similar analysis of the liver proteome (Fig. 5B) showed a trajectory in mCAT aging that was similar to that of WT aging, although the slope was slightly lower. As noted earlier, this smaller effect is likely explained by the lack of detectable mCAT expression in liver tissues.

### Changes in major proteostasis pathways

**Figure 6:**
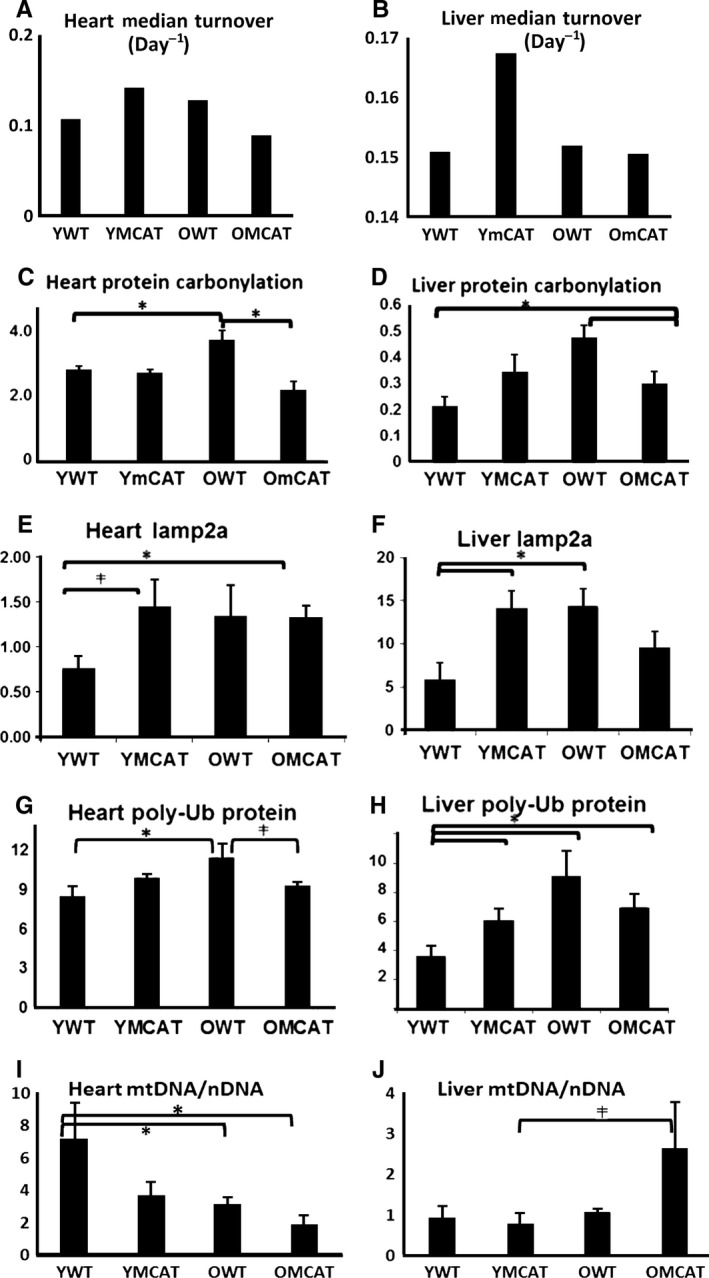
mCAT and aging alter markers of several protein turnover mechanisms. Western blotting was performed on various markers of turnover pathways and compared to the median global protein turnover rates determined by mass spectrometry in (A) heart and (B) liver. (C) Protein carbonylation increased with age and was attenuated in old mCAT in heart. (D) Liver tissue similarly increased in carbonylation with age, and this was attenuated in old mCAT mice. (E) Chaperone‐mediated autophagy, as measured by lamp2a, was increased in both young and old mCAT hearts. (F) Lamp2a was elevated in YmCAT as well as in OWT mouse liver. (G) Levels of polyubiquitinated proteins significantly increased with age in the heart, and this effect was attenuated in old mCAT. (H) Levels of polyubiquitinated proteins in the liver were increased by both mCAT and aging. * *P*‐value < 0.05.

Median turnover rates, which were calculated directly from median half‐lives (Fig. 1A) and plotted as bar graphs for each experimental group in heart (Fig. 6A) and liver (Fig. 6B), suggest that global turnover may be highest in YmCAT and OWT and lowest in YWT and OmCAT mice. To explore some of the mechanisms by which abundance and turnover might be affected by mCAT and aging, we measured markers of autophagy, oxidation, the ubiquitin proteasome, mitochondrial biogenesis, and protein translation (Fig. 6C–H, Fig. S7C–F, Fig. S8A–H). Oxidation of proteins, as measured by protein carbonylation, was increased with age in WT hearts (Fig. 6C) and liver (Fig. 6D), as expected. This increase was not seen (heart) or was smaller in old mCAT mice and carbonylation remained unchanged in young mCAT mice. In concordance with faster protein turnover in YmCAT tissues, lamp2a levels, a marker of chaperone‐mediated autophagy, were increased in YmCAT hearts and livers to levels similar to that seen in old WT tissues (Fig. 6E,F). Levels of polyubiquitinated proteins were increased in OWT hearts and in all groups of liver relative to YWT (Fig. 6G,H). This is consistent with the increases seen in the ‘protein ubiquitination’ pathway of the proteomic dataset.

The p70s6k signaling pathway, consisting of ribosomal proteins, elongation factors, and initiation factors, all of which positively regulate protein translation, showed significantly increased protein expression in old WT and young mCAT mice in the pathway analysis, a pattern consistent with the increased global protein turnover rates seen in these groups. We confirmed these increases in eEF2 by Western blot (Fig. S8A,B). Macroautophagy was measured by the ratio of LC3II/I to Beclin1 (Fig. S8C,D). The LC3II/I ratio was not significantly altered in any group. However, these markers cannot determine flux through the lysosomal pathway, and therefore, this would require further study.

To determine whether mitochondrial biogenesis was significantly altered, we performed analysis of mitochondrial DNA copy number (Fig. 6I,J) and Western blotting for PGC1α and COXIV (Fig. S7C–F). There was a significant decrease in mitochondrial DNA copy per cell in old WT and old mCAT hearts (Fig. 6I), but a trend toward increased mito copy number in the OmCAT liver (Fig. 6J), suggesting mitochondrial copy number may account for some of the protein abundance differences. There were no differences in PGC1α in either tissue.

## Discussion

This study presents a comprehensive characterization of proteome dynamics during aging in wild‐type and long‐lived mCAT mouse hearts and livers. To determine *in vivo* proteome turnover kinetics and protein abundance, we utilized a metabolic labeling strategy in combination with LC‐MS/MS and Topograph software. We were surprised to find that mCAT has very different effects in young compared with old mouse hearts, with YmCAT mice resembling OWT, in addition to OmCAT hearts having a more ‘youthful’ proteome. This effect was observed in two independent datasets.

We observed globally decreased protein half‐lives with age in both heart and liver, as previously reported in liver (Karunadharma *et al*., 2015), although this change did not reach a statistical significance in the previous report in heart (Dai *et al*., 2014). Changes in HL in WT aging in both heart and liver within individual pathways were concordant with these prior observations, including the pathways of mitochondrial dysfunction, branched chain amino acid metabolism, actin cytoskeleton, oxidative stress response, ethanol degradation, and aryl hydrocarbon receptor signaling. Likewise, changes in protein abundance during WT aging were significant in most of the same pathways as previously reported. In heart, this included mitochondrial dysfunction, actin cytoskeleton, calcium signaling, and LXR/RXR activation (Dai *et al*., 2014). In liver abundance, this included EIF2 signaling, p70S6K, and LPS‐ and IL1‐mediated RXR function (Karunadharma *et al*., 2015). A large and consistent finding in the present study and our previous reports (Dai *et al*., 2014; Karunadharma *et al*., 2015) is the observation of a lengthening of median half‐lives of the global proteome by the addition of an aging intervention, as previously reported for calorie restriction (CR) and rapamycin treatment. This may seem at odds with the fact that both CR and rapamycin have been shown to induce autophagy (Laplante & Sabatini, 2012); however, we have recently shown that this is a transient effect in mice, seen at 1–2 weeks post‐treatment (Chiao *et al*., 2016). In parallel with decreased protein turnover, we also observed reductions in protein carbonylation and total protein ubiquitination, surrogate markers of protein damage and unfolding (Nyström, 2005; Kastle & Grune, 2011; Cuanalo‐Contreras *et al*., 2013), in old mCAT (Fig. 6), CR and rapamycin (Dai *et al*., 2014)‐treated mice relative to old WT mice. Likewise, it has been shown that constitutive activation of TORC1 decreased translational fidelity and increased amino acid misincorporation errors and that rapamycin reversed these effects as it slowed the rate of ribosomal elongation in mouse embryonic fibroblasts (Conn & Qian, 2013). In the context of limited nutrient availability, it makes sense to preserve the proteome and limit the production of new proteins. The reason for longer proteome half‐lives in old mCAT mice is less clear, although one possibility may be that reduction in mtROS is a common component of all of these interventions that is sufficient to reduce protein damage and thereby improve quality and increase half‐lives. ROS is also a powerful signal for cellular energy status and is closely tied to the allocation of cellular resources. It can signal synthesis and degradation of proteins as well as cellular fate, not unlike the nutrient‐sensitive mammalian target of rapamycin (mTOR) pathway (Sarbassov & Sabatini, 2005; Squier, 2006), and may therefore utilize some similar mechanisms to increase proteome half‐lives and promote longevity. These observations raise the question of whether enhanced protein quality and reduced protein turnover is a common underlying longevity mechanism in these and perhaps other interventions. This has recently been proposed in a study of protein half‐lives across multiple longevity interventions (Thompson *et al*., 2016).

One aspect of the mCAT proteome, however, is novel compared to other interventions thus far: an appearance of antagonistic pleiotropy. While old mCAT mice are benefited with a more youthful pattern of proteome half‐life and abundance, corresponding with longer life (Schriner *et al*., 2005) and numerous healthspan benefits (Dai *et al*., 2009, 2013; Lee *et al*., 2010a; Mao *et al*., 2012), the young mCAT proteome assumes an ‘older’ phenotype in a young animal, in both protein abundance and half‐life. Antagonistic pleiotropy is typically seen as an effect that is beneficial to an organism's fitness early in life, but which causes functional decline and aging phenotypes later in life. In this case, we observe the opposite in mCAT mice. Hence, this effect, illustrated in Fig. 5, appears consistent with reverse antagonistic pleiotropy.

At moderate or low levels, ROS are increasingly being found to serve important physiological signaling roles that may be important for metabolism, protein turnover, cellular differentiation, stress response, and apoptosis (Starkov, 2008; Reczek & Chandel, 2015), while damaging effects may not be apparent until ROS reaches high levels. For example, low‐level ROS is important for the renewal of stem cells, and it has been shown that suppression of ROS significantly limits the renewal of stem cells and suppresses neurogenesis in mice (Le Belle *et al*., 2011). Thus, overexpression of mitochondrial‐targeted catalase might lead to similar effects. In fact, a recent study has reported that while low levels of transgenic mCAT expression were beneficial in a cardiac Mfn2‐knockout background, ‘supersuppression’ of ROS by higher levels of mCAT exacerbated the cardiac phenotype and suppressed compensatory autophagy (Song *et al*., 2014). A separate study found that bactericidal activity is impaired in young mCAT mice (West *et al*., 2011). It has also been proposed that ROS at low levels has a direct beneficial effect as part of a process termed mitohormesis (Ristow & Zarse, 2010). At high levels, ROS cause oxidative damage, such as in cardiac hypertrophy and failure, and in this setting, high and low levels of mCAT are both protective (Dai *et al*., 2011). In the present study, the proteomic changes from mCAT overexpression in unstressed, young animals mimic a number of changes that occur with aging. Taken together, these studies suggest that mitochondrial antioxidant may not be universally beneficial, and the beneficial effects are observed in a setting when oxidative stress or a high burst of ROS is anticipated. Thus, as with many drugs, mitochondrial antioxidants likely have a therapeutic windows and this may be age dependent. It is also possible that such therapeutic windows vary by genetic background and organism.

These observations are also consistent with the notion of a ROS continuum, at the center of which is a physiologically necessary level of mitochondrial ROS (Marcinek & Siegel, 2013) (Fig. 5D). At lower extremes of the continuum, redox signaling pathways may be impaired, while at higher extremes ‘pathological’ ROS damages important components of the cell. In aging, increasing levels of ROS and oxidative damage are widely documented and were part of the support for the ‘free radical theory of aging’ (Shi *et al*., 2010). However, the mCAT effect in young mice is consistent with an adverse impact on the more beneficial effects of ROS, while mCAT suppression of pathological levels of ROS in old animals is protective. This also suggests that ROS itself exhibits conventional antagonistic pleiotropy and would explain why stronger antioxidant mechanisms, such as mCAT, have not evolved under natural selection of young animals in nature.

It would be of interest to pursue assays of reproductive fitness in mCAT vs. WT mice as a further test of the hypothesis of reverse antagonistic pleiotropy. We have not detected such an effect in the laboratory; however, such tradeoffs in fitness may only be present in natural environments. Alternatively, one could pursue surrogate functional assays, such as tests of fighting behavior or endurance on treadmills (Torma *et al*., 2014). Another area for future research would be to examine additional ages of mCAT and WT mice to test the unproven assumption that there is an approximately linear decline in the youthful status of the proteomes over the life course.

In conclusion, we show evidence of ‘reverse’ antagonistic pleiotropy in the cardiac and hepatic proteomes of mice overexpressing mitochondrial‐targeted catalase. In old mice, mCAT overexpression leads to a youthful proteome and better health, while in young mice it confers an ‘older’ proteome. These data support a view of ROS in aging that is inclusive of the beneficial functional roles ROS plays, such as redox signaling, in addition to pathological effects of ROS‐mediated oxidative damage. Importantly, these data underscore the importance of dose and age with respect to therapeutic interventions. In particular, in order for mitochondrial antioxidants to have a desired effect in humans, it may be important not only to consider the correct dose, but also to adjust the dose (or any delivery at all) with age.

## Materials and methods

### Animals

We used two independent founder lines as described before (Schriner *et al*., 2005); B6.C3H‐Tg(mCAT)4033Wcl and B6.C3H‐Tg(mCAT)4403Wcl were generated and back‐crossed as hemizygotes onto the C57BL/6J background (Jackson Laboratories, Bar Harbor, ME) for more than ten generations. Control animals were wild‐type (WT) littermates of MCAT transgenic animals. Mice were started on a synthetic diet (Harlan Teklad diet #TD.99366) that was nutritionally similar to the NIH‐31 standard for rodents. The use of this diet facilitated the subsequent substitution of heavy‐labeled [5,5,5‐^2^H_3_] leucine for light leucine, which enabled the protein turnover measurements.

### Stable Isotope labeling

After 10 weeks of diet regimens, all mice received a synthetic diet (TD.09846, Harlan Teklad, Madison, WI) with the light leucine fully replaced by 11 g/kg of deuterated [5,5,5‐^2^H_3_]‐L‐leucine (Cambridge Isotope Laboratory, Tewksbury, MA), with CR and RP cohort conditions continued as above. Three mice were euthanized for tissue collections and proteomics analysis at four time points: days 3, 7, 12, and 17 after switching to ^2^H_3_‐leucine diet.

### Mass spectrometry

Tissues were homogenized in cold isolation buffer (250 mm sucrose, 1 mm EGTA, 10 mm HEPES, 10 mm Tris–HCl pH 7.4). These lysates were centrifuged at 800 × *g* for 10 min to get rid of the debris. Whole liver and heart tissues were homogenized and trypsin‐digested, and LC‐MS/MS analysis was performed with a Waters nanoAcquity UPLC and a Thermo Scientific LTQ Orbitrap Velos, as previously described (Hsieh *et al*., 2012).

### Data repository

The raw data from MS/MS and extended supplementary files are available at https://chorusproject.org/pages/blog.html#/678. To view the data, a free account must be obtained by following the instructions on the Chorus Project Web site. For R‐scripts, spreadsheets, and other data, please contact the corresponding author.

### MS data analysis

MS data were processed with the Hardklor (v1.33) and Bullseye (v1.25) algorithms to refine precursor mass measurements, followed by database search against all mouse entries of the UniProt database (UniProt release 2013_02) with the SEQUEST algorithm (vUW2012.01.7)—searching a total of 74 888 protein entries that were designated Mus musculus (Mouse). A dynamic modification of 3.0188325 for leucine was set to account for [5,5,5‐^2^H_3_]‐leucine, and a static modification of 57.021461 for cysteine was set for carbamidomethyl modifications. The precursor monoisotopic mass tolerance was set to ± 10 ppm, and the fragment mass tolerance window was set to 0.36 m/z. Enzyme specificity was set to semi‐tryptic, allowing for up to two missed cleavage sites per peptide. The false discovery rate for spectrum matches was determined by the Percolator algorithm (v2.04) using a reversed copy of the UniProt database as a decoy. Only results with a *q*‐value less than 0.01 were kept for further analysis. This filter was used as it allows a small number (1%) of false positives to the next portion of analysis while providing enough data for categorization and statistical analysis of subsets of proteins. In addition, less than 5% of accepted false positives are expected to pass a subsequent turnover score filter, described below.

Topograph (v1.1.0.297) software was developed for the deconvolution and measurement of peptide isotopologue abundance from LC‐MS chromatograms and the calculation of peptide turnover rates, as previously described (http://proteome.gs.washington.edu/software/topograph/). Prior to analysis of abundance and turnover, peptides with a turnover score less than 0.98 were filtered out of the data. Turnover score is a metric internal to Topograph, ranging from 0 to 1, which describes the closeness of each observed isotopologue distribution with its closest matching theoretical distribution. A cutoff score of 0.98 was derived by plotting a receiver operator curve of true‐positive results vs. true‐negative results (not shown), where true positive was defined as any peptide measurement that fell within two standard deviations of the mean label enrichment for all peptides and true negatives were results that did not meet these criteria. Relative peptide abundance was determined by integrating MS1 peaks. For peptides that were identified in one sample, the regression of the identified peptide's MS/MS scan number is used to estimate a window for the same peptide in the other samples and a matching chromatographic peak was identified within that time range. This method allows peaks areas to be measured even in samples in which they are low and otherwise difficult to identify.

Only peptides that uniquely mapped to a single UniProt protein accession for Mus Musculus (UniProt release 2013_02), consisting of 74, 888 entries from UniProtKB/*Swiss*‐*Prot* and UniProtKB/*TrEMBL*, were used for the quantification of abundance and turnover. To map peptides to proteins, peptide sequences were searched against the sequences of all proteins in the Swiss‐Prot database, and kept if a unique match was found. If no match was found, a second search was performed on TrEMBL entries and the unique matches were retained. All remaining peptides, consisting of peptides with either no matching proteins or greater than 1 matching protein, were filtered out. For the cases where a protein consisted of more than one peptide, statistical models were modified to appropriately account for the multiple peptides by using a blocking factor. For each protein, we applied nonlinear regression fits of first‐order exponential curves to the percent newly synthesized protein using y = 100 + β1^eαt^. To determine whether the rates of turnover (slopes, α) were statistically different between experimental groups, ANCOVA was used. Half‐lives are calculated directly from slopes, where t_1/2_ = ln/slope. For details, see the methods supplement of Hsieh *et al*. (2012).

For heatmaps and pathway enrichment, only proteins that had significantly changed (*P*‐value < 0.05) with age (significantly different between YWT and OWT) were considered. The *P*‐values and correlations of the bivariate plots in panels B and C of Figs 4 and S4 were derived from a partial correlation of the plotted groups while controlling for covariance with young wild‐type samples. Partial correlation allows direct comparison of peak areas (abundance) while controlling for changing baseline intensity caused by peptide variation in ionization efficiency. The YWT treatment group was used as the baseline for all other groups. Heatmaps were created using the heatmap.2 function in the gplots package in R. Rows and columns were ordered by linkage clustering using a Euclidean distance measure.

Line plots displayed in Fig. 5 were calculated using proteomic abundance values (peak areas) of all proteins that significantly changed in abundance with age below a *P*‐value threshold of 0.05. To compare the trajectories of wild‐type aging and mCAT aging, we condensed the proteomic changes into an index of the aging change by taking the average absolute magnitude of the fold changes in protein abundance from YWT to OWT (WT aging). By this metric, WT aging is an average 5.66 fold change in heart proteome abundance. YWT was then set to zero, and all values are expressed as a percentage of the WT aging effect; thus, the 5.66 fold change in OWT equals 100% (black line, Fig. 5A). Young and Old mCAT were adjusted accordingly such that all differences from YWT in the same direction as aging were positive values and changes in the opposite direction were negative values. The mCAT aging line was then plotted by connecting two points representing YmCAT and OmCAT.

### Pathway analysis

Top pathways were determined using QIAGEN'S^®^ Ingenuity Pathway Analysis (IPA^®^, QIAGEN Redwood City, www.qiagen.com/ingenuity) on all proteins which were significantly changed (*P*‐value < 0.05) in abundance or half‐life by aging (OWT vs YWT) or mCAT (YmCAT vs YWT and OmCAT vs OWT) expression. IPA determines the *P*‐values of enrichment into canonical pathways by Fischer's exact test. All significantly changed proteins were then grouped by IPA canonical pathway, and z‐scores were visualized on a heatmap created in R using the gplots package. Values within each pathway category were clustered by Ward's method.

### Immunoblotting and ELISA

A portion of each tissue was aliquoted into separate tubes containing cold isolation buffer (250 mm sucrose, 1 mm EGTA, 10 mm HEPES, 10 mm Tris–HCl pH 7.4) as well as protease and phosphatase inhibitors (Pierce #87786 and #78420, Waltham, MA, USA) at the time of harvest and stored at −80 °C until used for immunoblotting and other bench assays as necessary. Western blotting was performed on the NuPAGE^®^ Bis‐Tris gel system (Life Technologies #WG1403BOX, Carlsbad, CA, USA) according to the manufacturer's protocols with primary antibody concentrations of 1000× and secondary antibody concentrations of 10 000×. The following primary antibodies were used: rabbit anti‐human erythrocyte catalase (Athens Research & Technology #01‐05‐030000, Athens, GA, USA), UQCRC2 (Abcam #ab103616, Cambridge, UK), VDAC (Thermo Scientific #PA1‐954A, Waltham, MA, USA), Lamp2a (Invitrogen #51‐2200, Waltham, MA, USA), mouse anti‐mouse multiubiquitin (MBL #D058‐3, Wooburn, MA, USA), eEF2 (Santa Cruz Biotechnology #sc‐166415, Dallas, TX, USA), LC3II/I (Cell Signaling #4108, Denver, MA, USA), Beclin1 (Cell Signaling #3495, Denver, MA, USA), PGC1a (Abcam #ab54481, Cambridge, UK), COX‐IV (Abcam #ab14744, Cambridge, UK). The following secondary antibodies were used: donkey anti‐rabbit, goat anti‐mouse, and rabbit anti‐goat. Protein carbonylation was measured using an OxiSelect^™^ Protein Carbonyl ELISA Kit (Cell Biolabs, Inc. #STA‐310, San Diego, CA, USA).

### Mitochondria Copy Number and mCAT gene expression

To quantify mitochondria copy number per cell, we isolated DNA from hearts and livers using a PureLink^®^ Genomic DNA Mini Kit, as per the manufacturer's instructions (Invitrogen K1820‐02, Waltham, MA, USA). Mitochondrial DNA copy number was quantified using quantitative PCR of total DNA extracts from cardiac tissues. Mitochondrial DNA copies were estimated by the ratio of the amount of mitochondrial gene NADH dehydrogenase 1 (ND1) and a single‐copy nuclear gene cytochrome P4501A1 (cyp1A1). Primers used were as follows: ND1 (For: GAACGCAAAATCTTAGGGTACATACA, Rev: GCCGTATGGACCAACAATGTT, probe: 6FAM‐CTACGAAAAGGCCC) and cyp1A1 (For:GACACAGTGATTGGCAGAGATC, Rev:AACGGATCTATGGTCTGACCTGT, probe: 6FAMCTCAGCTGCCCTATCTGGAGG CCTTC). Mitochondrial catalase qPCR was performed using forward and reverse primer sets for human catalase.

## Author Contributions

NB, PSR and GMM. wrote the paper. NB, DFD, MJM and PSR designed the study and developed the methodology. NB, DFD, AG, JF, LG and YM processed samples for proteomics and performed bench experiments. RPB and MJE assisted in proteomic analysis and statistical analysis. EJH assisted in sample preparation and performed MS data acquisition.

## Funding

No funding information provided.

## Conflict of interest

The authors declare no conflict of interest.

## Supporting information


**Fig. S1** Experimental workflow for metabolic labeling and proteomic analysis.

**Fig. S2** Histograms of changes in peptide abundance during 17‐days of Heavy Leucine diet.

**Fig. S3** Density plot of half‐life (in days) in heart and liver tissue.

**Fig. S4** Heatmap containing hepatic protein abundance changes.

**Fig. S5** Western blotting and qPCR of mCAT expression in hearts and livers.

**Fig. S6** Earlier studies of proteomic abundance independently confirm a pattern of antagonistic pleiotropy.

**Fig. S7** Western blotting of mitochondrial proteins and markers of mitochondrial biogenesis.

**Fig. S8** Western blotting of markers of protein synthesis and degradation.

**Data S1** Spreadsheets relative abundance statistics and turnover statistics in the cardiac proteome.

**Data S2** Spreadsheets relative abundance statistics and turnover statistics in the hepatic proteome.
