# Supplementary figures and images for: Mitochondrial‐targeted catalase is good for the old mouse proteome, but not for the young: ‘reverse’ antagonistic pleiotropy?
Source: Aging Cell. 2016 Apr 8;15(4):634–45. doi: 10.1111/acel.12472 (PMC4933659; doi:10.1111/acel.12472)

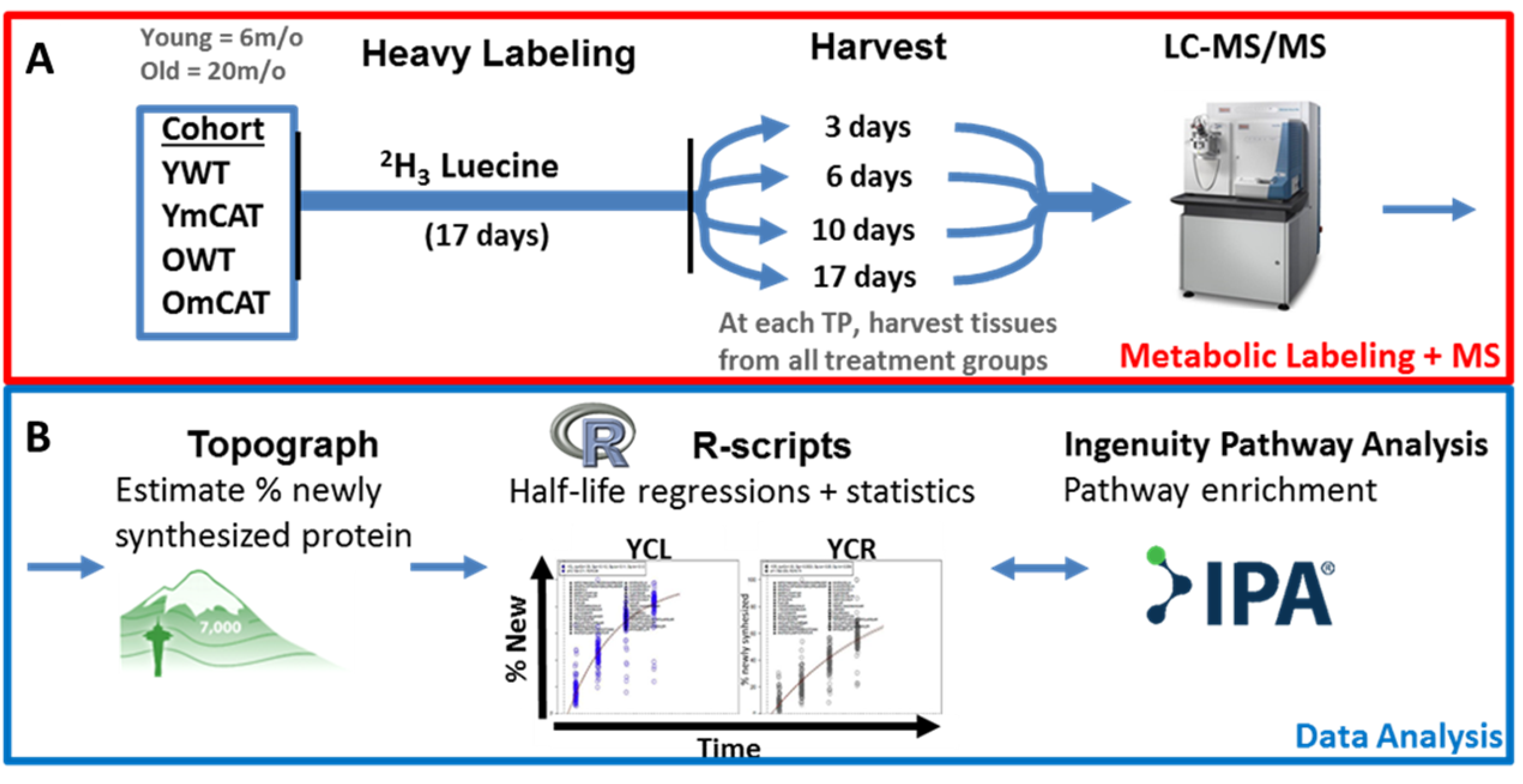

Supplement: Supplementary file 1 — Fig. S1 Experimental workflow for metabolic labeling and proteomic analysis. [file ACEL-15-634-s001.tiff]

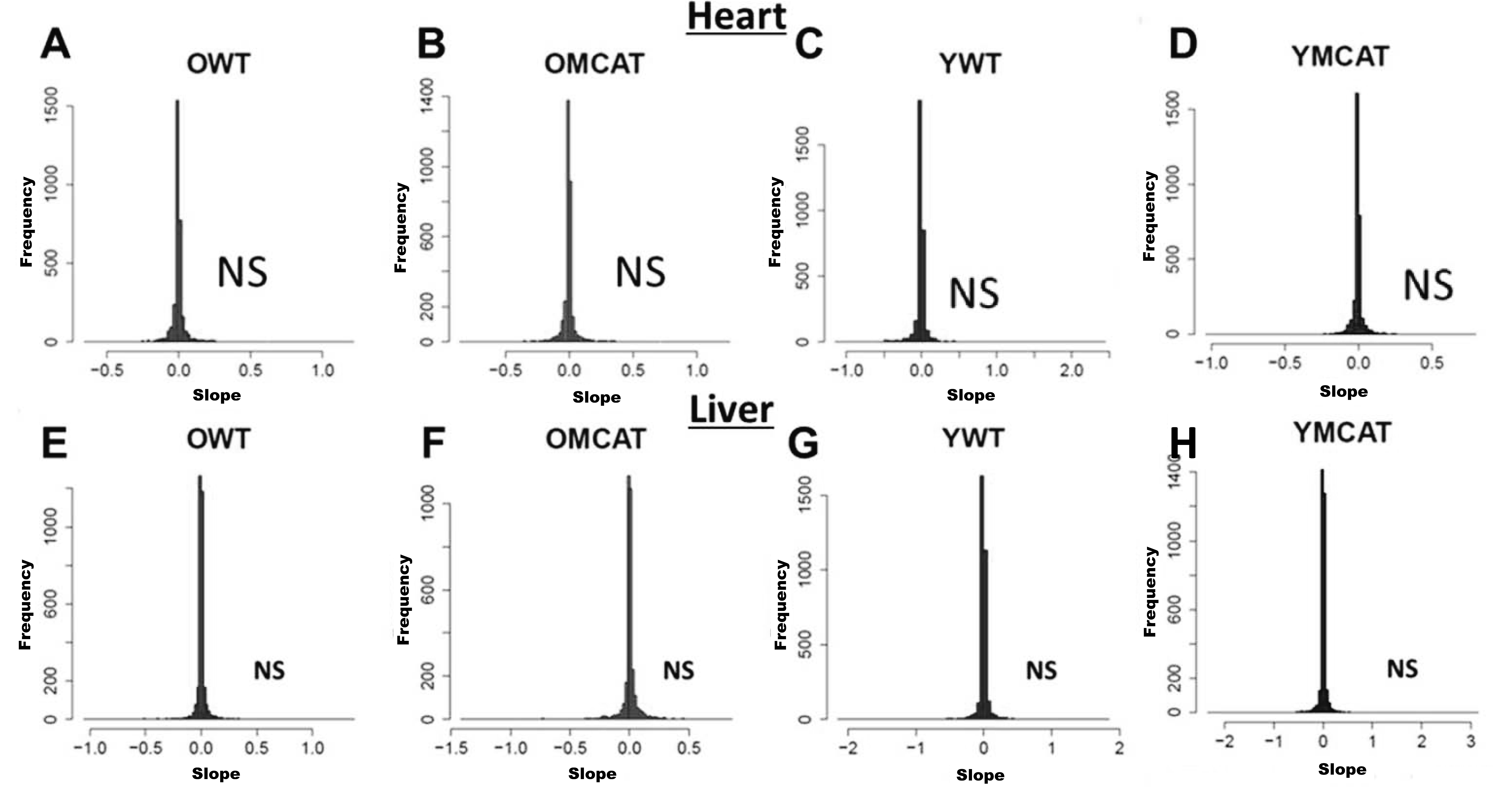

Supplement: Supplementary file 2 — Fig. S2 Histograms of changes in peptide abundance during 17‐days of Heavy Leucine diet. [file ACEL-15-634-s002.tiff]

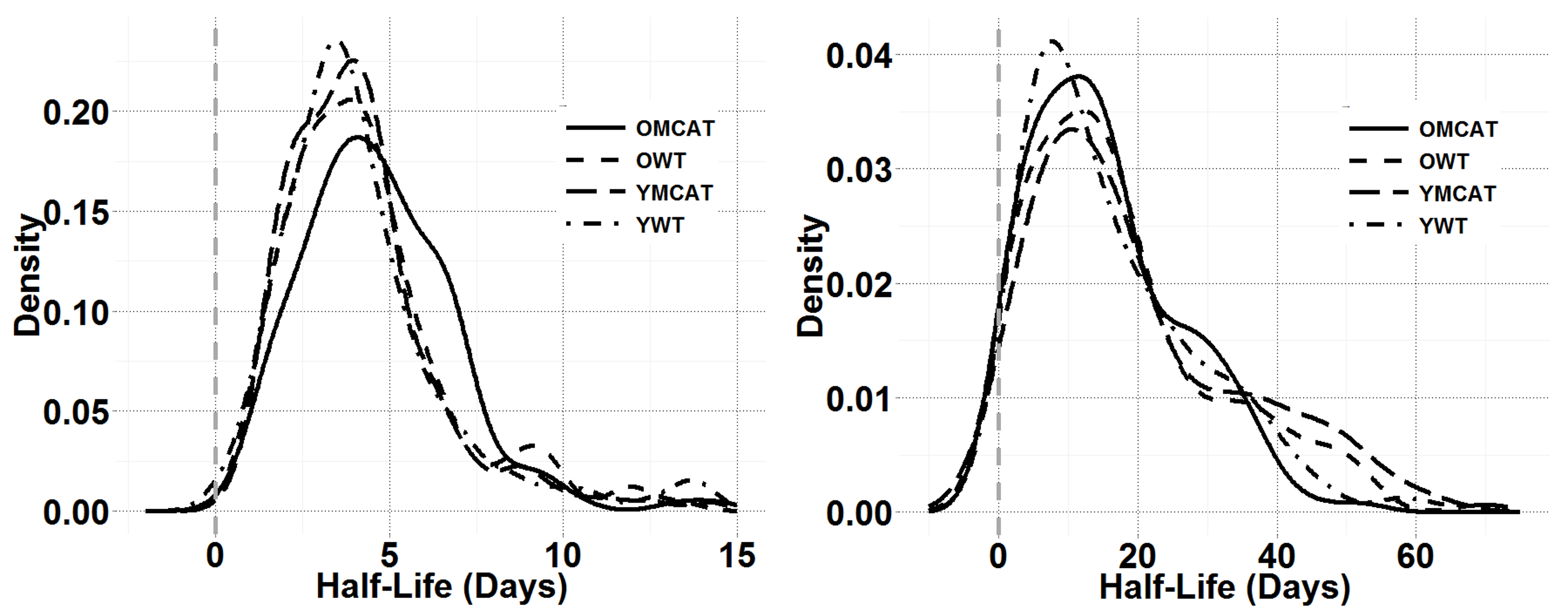

Supplement: Supplementary file 3 — Fig. S3 Density plot of half‐life (in days) in heart and liver tissue. [file ACEL-15-634-s003.tiff]

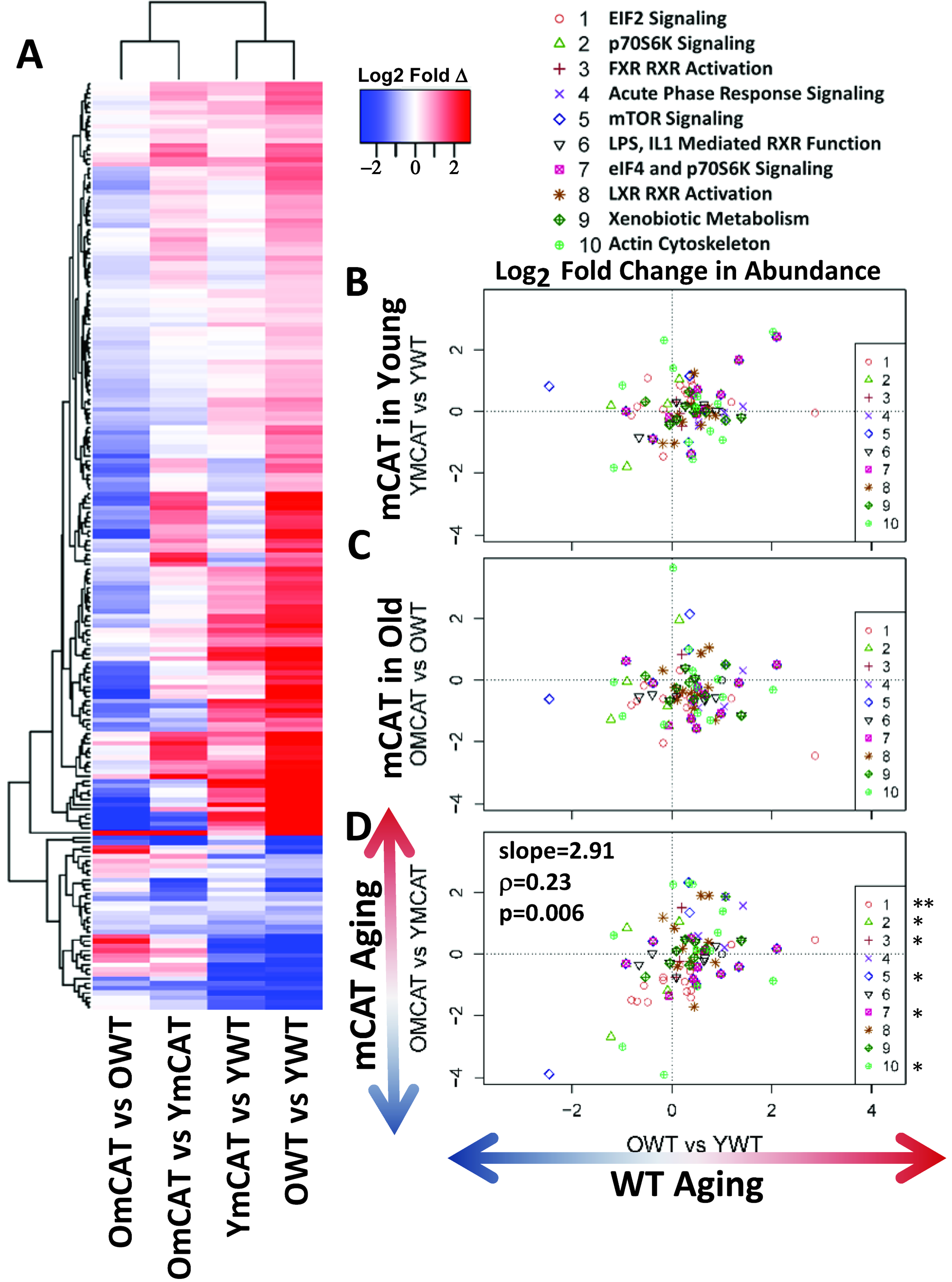

Supplement: Supplementary file 4 — Fig. S4 Heatmap containing hepatic protein abundance changes. [file ACEL-15-634-s004.tiff]

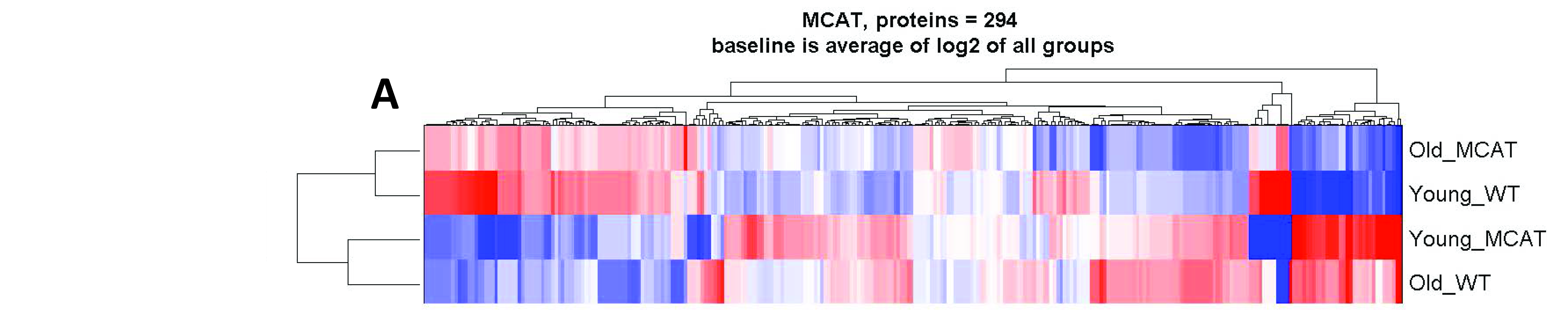

Supplement: Supplementary file 6 — Fig. S6 Earlier studies of proteomic abundance independently confirm a pattern of antagonistic pleiotropy. [file ACEL-15-634-s006.tiff]
