# Supplementary material for: Mitochondrial‐targeted catalase is good for the old mouse proteome, but not for the young: ‘reverse’ antagonistic pleiotropy?
Source: Aging Cell. 2016 Apr 8;15(4):634–45. doi: 10.1111/acel.12472 (PMC4933659; doi:10.1111/acel.12472)
Supplement: Supplementary file 9 [file ACEL-15-634-s009.docx]

**Supplementary Figure Legends**

**Supplementary Figure 1: Experimental workflow for metabolic labeling and proteomic analysis.** A) YWT, YmCAT, OWT, and OmCAT mice were placed on a diet in which leucine was substituted for deuterated leucine for up to 17 days. At each of four timepoints – 3, 7, 12, and 17 days – mice from each of the four groups were sacrificed, tissues harvested, and flash frozen until used to prepare total lysates. Total lysates from hearts and livers were subject to shotgun (data-dependent) mass spectrometry analysis. B) Topograph software was used to estimate the percentage of newly synthesized protein for every protein at each timepoint, as well as area under the curve for each peptide. First-order exponential regressions of the percentage of newly synthesized protein over time were performed in R to determine half-lives. Abundances, as determined by peptide peak areas, and half-lives for each experimental group underwent statistical comparisons and visualization in R. Ingenuity Pathway Analysis software was used to perform pathway enrichments into top canonical pathways.

**Supplementary Figure 2: Histograms of changes in peptide abundance during 17-days of Heavy Leucine diet.** Over the 17-day labeling period an increasing proportion of peptides contains deuterated leucine, therefore it is increasingly important to quantify abundances based on both the unlabeled and labeled peaks formed by every peptide. We determined the slope of a line fitted through the peak area of every peptide over the 17-day labeling period, depicted here as histograms for heart (A-D) and liver (E-H). There was no difference between the mean of these slopes and zero, as measured by a two-tailed t-test.

**Supplementary Figure 3: Density plot of half-life (in days) in heart and liver tissue.** The half-lives of all proteins detected in A) heart and B) liver tissues are shown.

**Supplementary Figure 4: Heatmap containing hepatic protein abundance changes** enriched into proteins significantly altered by aging (p-value< 0.05) and correlation plots of mCAT versus WT aging effects. A) The heatmap shows the direction and magnitude of abundance changes between the groups written in the column labels. B-D) Correlations were done on the top 10 pathways to compare the effects of mCAT to the effects of WT aging (OWT vs YWT). B) The effect of mCAT in young mice (YmCAT vs YWT) is significantly correlated with WT aging changes, but to a lesser extent than heart tissue. C) The effect of mCAT in old mice (OmCAT vs OWT) has no relationship with WT aging (OWT vs YWT), D) mCAT aging (OmCAT vs YmCAT) is significantly but weakly correlated with normal aging (OWT vs YWT). ***** p- value < 0.05, ** p- value < 0.001 for Spearman correlation of the individual pathway between the x and y-axis groups.

**Supplementary Figure 5: Western blotting and qPCR of mCAT expression in hearts and livers.** Expression of mCAT on both the protein and transcriptional level were able to detect mCAT expression in the hearts, but not livers, of transgenic animals. The same mice were used for both assays.

**Supplementary Figure 6: Earlier studies of proteomic abundance independently confirm a pattern of antagonistic pleiotropy.** A heatmap is shown which depicts the results of an initial large-scale survey of abundance and turnover changes in WT and mCAT mice with age.

**Supplementary Figure 7: Western blotting of mitochondrial proteins and markers of mitochondrial biogenesis**. Western blotting of A) Uqcr2 and B) VDAC confirm changes seen in these proteins in the heart proteome. To examine mitochondrial biogenesis and content we measured the marker PGC1alpha in C) heart and E) liver. COX-IV was also measured in D) heart and F) liver as a proxy for mitochondrial content.

**Supplementary Figure 8: Western blotting of markers of protein synthesis and degradation.** The elongation factor eEf2, a marker of translation, was measure in A) hearts and B) livers. Macroautophagy was examined with C-D) the ratio of LC3II/I and E-F) Levels of Beclin1. G-H) Chaperone-mediated autophagy was measured by the lysosomal receptor Lamp2a.
